# Supplementary material for: The Cinderella Complex: Word embeddings reveal gender stereotypes in movies and books
Source: PLoS One. 2019 Nov 22;14(11):e0225385. doi: 10.1371/journal.pone.0225385 (PMC6874350; doi:10.1371/journal.pone.0225385)
Supplement: S1 File — We analyze overall emotion, negative emotion, a life unpacked during the co-occurrence, controlling story genres and periods and robustness check of regression models. (DOCX) [file pone.0225385.s009.docx]

## **Overall Emotion**

To rule out the possibility that the higher increase in happiness for female characters maybe not due to the presence of male characters, but to the overall tendency of describing female characters with more emotional words, we compare the happiness score when females and males are present in the same context with that when they are not.

We calculate the happiness score averaged over the whole course of stories (i.e., overall emotion) for both males and females, and compare the group difference (see S1 Fig). We find that the overall happiness level of female characters is higher than male characters. This finding supports the overall tendency of describing female characters with more emotional words.

In S2 Fig, we compare females’ happiness score when they encounter males and that when they do not encounter males, and find that the happiness level of female characters increases significantly with the presence of male characters. Therefore, the between-group difference we find in S1 Fig does not explain away the within-group variance we observed--females are happier when they encounter males in S2 Fig.

## **Negative Emotion**

We have analyzed the increase in happiness in Fig 4 of the manuscript. It is also necessary to analyze the decrease in happiness. As S3 Fig shows, the decrease in happiness, conditional on the co-occurrence with the other gender, is higher for female than for male characters. These findings suggest that females are more emotionally dependent on males in both directions. But this result does not influence our primary finding: females are happier when they encounter males, no matter compared against the other gender group (Fig 4) or compared within the group between two states (encounter or not, see S2 Fig).

## **A Life Unpacked During the Co-Occurrence**

Aside from analyzing all the words describing the male and female characters (Figs 5-7), we also select and analyze the words within the context of the presence of both genders. The observed difference between groups in Figs 5-7 remains significant (S4-S6 Figs).

Within each of the 6,087 movie synopses, we select five words before and five words after the names of the leading characters across all the sentences containing both names of the female and male leading characters. We iterate over the pairwise combinations of words within 10-word samples across all movie synopses to construct word co-occurrence networks, one for females and the other for males (S4-5 Figs). We then identify communities from these two networks using the Q-modularity algorithms [35]. Three communities emerge from the female network, including action, family, and romance. And five communities are identified from the male network, including action, family, romance, crime, and career. Compare with Fig 5, this community structure reveals males characters could be more career-oriented especially when they encounter females.

We further cut both networks into three slices by word categories, including adjectives, verbs, and nouns (S5 Fig). The differences in the distribution of words portray stereotypical gender images in detail. Further, we observed that male characters are more likely than female characters to be described using verbs across three datasets (S6 Fig).

**Controlling Story Genres and Periods**

We analyze three datasets, including 6,087 movie synopses (**a**), 1,109 movie scripts (**b**), and 7,226 books (**c**). The increase in happiness conditional on the co-occurrence with the other gender across different times (S7 Fig) and genres (S8 Fig), measured in the average of positive regression coefficients *k*, are shown as bars (blue for males and orange for females).

The increase in happiness, conditional on interaction with the other gender, is always stronger for females than for males. This finding suggests the existing stereotypes on the incompetence of female characters (Fig 4) are robust across story periods and genres (S7-S8 Figs). Further, we find that gender stereotypes are strengthened over years across our three datasets.

**Robustness Check of Regression Models**

We construct stepwise regression models to add one variable at a time and found that the number of co-occurrence and leading gender do not rule out the impact of happiness increase on the size and scale of movie rates.

**Table A. OLS Linear regression models predicting the quality of movie synopsis.** For p values, * denotes 0.01-0.05, ** denotes 0.001-0.01, and *** denotes <0.001.

|  | **Quality (rating)** | | |
| --- | --- | --- | --- |
| Constant | 6.10*** | 6.20*** | 6.23*** |
| Leading gender (male=1, female=0) | 0.18*** |  |  |
| N of co-occurrence |  | 0.08*** |  |
| Increase in happiness for the female | 0.04* | 0.08*** | 0.06*** |
| Increase in happiness for the male | -0.09*** | -0.09*** | -0.11*** |
| R squared | 0.014 | 0.012 | 0.010 |
| F-statistic | 15.36 | 13.16 | 15.90 |
| N of cases | 6,087 | | |

**Table B. OLS Linear regression models predicting the popularity of movie synopsis.** For p values, * denotes 0.01-0.05, ** denotes 0.001-0.01, and *** denotes <0.001.

|  | **Popularity (N of votes)** | | |
| --- | --- | --- | --- |
| Constant | 8.00*** | 8.29*** | 8.28*** |
| Leading gender (male=1, female=0) | 0.37*** |  |  |
| N of co-occurrence |  | 0.02*** |  |
| Increase in happiness for female | 0.02* | 0.12*** | 0.06* |
| Increase in happiness for male | -0.40*** | -0.32*** | -0.39*** |
| R squared | 0.04 | 0.04 | 0.03 |
| F-statistic | 41.85 | 48.87 | 56.11 |
| N of cases | 6,087 | | |
