# Supplementary material for: The Cinderella Complex: Word embeddings reveal gender stereotypes in movies and books
Source: PLoS One. 2019 Nov 22;14(11):e0225385. doi: 10.1371/journal.pone.0225385 (PMC6874350; doi:10.1371/journal.pone.0225385)
Supplement: Attachment — Submitted filename: Response to Reviewers.docx [file pone.0225385.s010.docx]

**Response Letter**

**Journal Requirements:**

**1. When submitting your revision, we need you to address these additional requirements.**

**Please ensure that your manuscript meets PLOS ONE's style requirements, including those for file naming. The PLOS ONE style templates can be found at**

**http://www.journals.plos.org/plosone/s/file?id=wjVg/PLOSOne_formatting_sample_main_body.pdf and** [**http://www.journals.plos.org/plosone/s/file?id=ba62/PLOSOne_formatting_sample_title_authors_affiliations.pdf**](http://www.journals.plos.org/plosone/s/file?id=ba62/PLOSOne_formatting_sample_title_authors_affiliations.pdf)

We reviewed and revised our manuscript to ensure it meets the journal requirements.

**2. Please ensure that you refer to Figures 4 and 8 in your text as, if accepted, production will need this reference to link the reader to the figure.**

We cited Figure 4 and Figure 8 in the current version.

**Additional Editor Comments:**

**Dear Authors,**

**Thank you very much for submitting your paper for publication with PLOS One. Both reviewers agree that the paper requires a major revision. Please read carefully and address all their comments.**

**Best regards,**

**Ilya Safro**

Dear Dr. Ilya Safro,

We are grateful for the opportunity to revise the manuscript in response to these thoughtful reviews, and we believe that the paper is much stronger for having incorporated the suggestions from the reviewers. We detail our responses and revisions as below.

**Reviewers' comments:**

**Reviewer's Responses to Questions**

**Comments to the Author**

**1. Is the manuscript technically sound, and do the data support the conclusions?**

**The manuscript must describe a technically sound piece of scientific research with data that supports the conclusions. Experiments must have been conducted rigorously, with appropriate controls, replication, and sample sizes. The conclusions must be drawn appropriately based on the data presented.**

**Reviewer #1: Partly**

**Reviewer #2: Yes**

**2. Has the statistical analysis been performed appropriately and rigorously?**

**Reviewer #1: No**

**Reviewer #2: Yes**

**3. Have the authors made all data underlying the findings in their manuscript fully available?**

**The PLOS Data policy requires authors to make all data underlying the findings described in their manuscript fully available without restriction, with rare exception (please refer to the Data Availability Statement in the manuscript PDF file). The data should be provided as part of the manuscript or its supporting information, or deposited to a public repository. For example, in addition to summary statistics, the data points behind means, medians and variance measures should be available. If there are restrictions on publicly sharing data—e.g. participant privacy or use of data from a third party—those must be specified.**

**Reviewer #1: Yes**

**Reviewer #2: Yes**

**4. Is the manuscript presented in an intelligible fashion and written in standard English?**

**PLOS ONE does not copyedit accepted manuscripts, so the language in submitted articles must be clear, correct, and unambiguous. Any typographical or grammatical errors should be corrected at revision, so please note any specific errors here.**

**Reviewer #1: Yes**

**Reviewer #2: Yes**

**5. Review Comments to the Author**

**Please use the space provided to explain your answers to the questions above. You may also include additional comments for the author, including concerns about dual publication, research ethics, or publication ethics. (Please upload your review as an attachment if it exceeds 20,000 characters)**

**Reviewer #1: The paper presents a computational analysis of gender stereotypes in movie synopses, movie scripts, and books from the project Gutenberg. Three types of analysis are performed:**

**1. Comparison of happiness changes for male and female characters when they interact;**

**2. The influence of happiness changes for male and female characters on movie quality and popularity;**

**3. Comparison of word usage in movies with the leading male or female character.**

Following the suggestions of the reviewer, we have included more statistical analysis to make sure that the findings are solid and sound. Please see the responses below, including the analysis of emotional changes over the whole course, more rigorous tests and more detailed explanation to results, etc. We believe the quality of this manuscript has been greatly improved, and we appreciate the reviewers for giving so many valuable suggestions.

***#1.1* The paper studies an important problem of gender stereotypes that persist over the years in English fiction books and movies. It presents several interesting analyses that shed light on the problem from different perspectives. However, the authors tend to over-generalize their conclusions. For example, the higher increase in happiness for female characters may be due not to the presence of a male character (“the Prince”), but to the overall tendency of describing female characters with more emotional words. The authors can compare the emotional changes (increases and decreases in happiness) for female and male characters over the whole course of the story (not only when the two characters interact). Similarly in Sec. 3 (Unpacking the lives of female and male characters), the authors can analyze all the words describing the male and female characters to discover the main clusters/topics/communities.**

We agree with reviewer #1 entirely that analyzing the overall tendency of female characters would strengthen the quality of our research. We find that although the overall happiness level of female characters is higher than male characters (S3 Fig), this between-group difference does not explain away the within-group variance we observed - the happiness level of female characters *significantly increases* with the presence of male characters (S1 Fig). We now include this analysis in the current version in the supporting information (S1 Fig, S3 Fig).

Besides, we follow the suggestions of reviewer #1 to analyze all the words describing the male and female characters (and not only the words describing their encountering). The difference between groups remains substantial (Figure 4, 5, 6). We include this analysis into the manuscript (Figure 4, 5, 6).

**S3 Fig. The Overall Happiness Level, is Higher for Female Than for Male Characters.** We analyze three datasets, including 6,087 movie synopses (**a**), 1,109 movie scripts (**b**), and 7,226 books (**c**). The happiness score averaged over the whole course of stories are shown as bars (orange for females and blue for males). The lines on the top of the bars show one standard deviation. Asterisks indict P values. * P ≤ 0.05, **P ≤ 0.01, *** P ≤ 0.001, and ns non-significant. The result is significant across the three datasets.

**S1 Fig. Females are Happier When They Encounter (Co-Occur in the Same Sentence) with Males**. We analyze three datasets, including 6,087 movie synopses (**a**), 1,109 movie scripts (**b**), and 7,226 books (**c**). Bars show the happiness scores, orange for co-occurring with males and blue otherwise. The lines on the top of the bars show one standard deviation. Asterisks indict P values. * P ≤ 0.05, **P ≤ 0.01, *** P ≤ 0.001, and ns non-significant. The result is significant across the three datasets.

**Figure 4. Word Co-Occurrence Networks Describing the Life Packages of Female vs. Male as Leading Characters.** For each of the 6,087 movie synopses under study, we select ten words surrounding the names of the leading characters (five words before and five words after) across all the sentences containing the names. We iterate over the pairwise combinations of words within each 10-word sample across all movie synopses to construct word co-occurrence networks, one for males and the other for females. The female network (**a**) has 39,284 nodes (words), and 921,208 links (pairwise combinations of words within samples) and the male network (**b**) has 46,909 nodes and 1,319,208 links. We detect communities from the networks using the modularity algorithm [[35]](https://paperpile.com/c/ynVwMM/AlS0). Top four communities emerge from both networks, including action, family, career, and romance in the female network and action, family, career, and crime in the male network. Only nodes of 1,500 or more links are labeled.

**Figure 5. The Distribution of Adjectives, Verbs, and Nouns in Female and Male Word Co-Occurrence Networks**. **a-c.** The distribution of adjectives (**a**, green labels), verbs (**b**, blue labels), and nouns (**c**, red labels) in the female word co-occurrence network as introduced in Figure 4a. **d-f.** The distribution of adjectives (**a**, green labels), verbs (**b**, blue labels), and nouns (**c**, red labels) in the male word co-occurrence network as introduced in Figure 4b. Word categories are detected using the Penn Treebank tagset [[36]](https://paperpile.com/c/ynVwMM/7v7r). Only nodes of 1,500 or more links are labeled.

**Figure 6. Males Use More Verbs Than Females.** We analyze three datasets, including 6,087 movie synopses (a), 1,109 movie scripts (b), and 7,226 books (c). For each movie synopsis, movie script, or book under study, we select ten words surrounding the names of the leading characters (five words before and five words after) across all the sentences containing the names. We detect word categories using the Penn Treebank tagset [[36]](https://paperpile.com/c/ynVwMM/7v7r) and calculate the probability of observing verbs, P(verb), across all 10-word samples for females or males within each dataset. The bars show the values of P(verb), blue for males and orange for females. The lines on the top of the bars show one standard deviation. Asterisks indict P values. * P ≤ 0.05, **P ≤ 0.01, *** P ≤ 0.001, and ns non-significant. The result is significant across the three datasets.

**Further, many details of the analyses need clarification:**

***#1.2* - How do you measure happiness for different characters (male and female) in the same sentence/paragraph where both characters appear (co-occurrence)?**

We regret that we did not explain this clearly, and we appreciate the comments from the reviewer, which provided an opportunity for us to explain our measure in detail. We now explain more in the current version in the method (Constructing the Happiness Vector and Calculating Happiness Scores) and the results (1. The Cinderella Complex: Men are Women’s Ways to Happiness).

For two characters in the same sentence, we assume that they share the same *raw* scores of happiness. But we also track the raw scores whenever there is only one character in the sentence. We then normalize the emotion scores within a character using the z-score method and plot the accumulated curve. This explains how the same raw scores from the sentences of both characters may contribute to different curves.

Take the story of *Cinderella* as an example (figure 1a), when Ella and Kit get married at the end of the story, they share the same *raw* happiness score estimated from the sentence. But for Ella, this score results in a more positive pull in the displayed curve than that of Kit, because Ella was upset of her stepmother before the marriage, which makes the raw happiness score from marriage translated into the higher z-score.

**#1.3 - Fig. 2-4: Do you take into account only positive slopes (as mentioned in P. 4)? Do both characters have to have a positive slope in emotional change for the text snippet to contribute to the average? Why don’t you consider negative slopes? Women can be in general described with more emotional words.**

We appreciate the advice from the reviewer, and we now compare both positive and negative slopes (S2 Fig). As predicted by the reviewer, females are more emotional than males in both directions (S2 Fig). We add this part into the current version. Please kindly note that this result does not influence our primary finding: females are happier when they encounter with males, whether compared against the other gender group (Figure 3), or within the group between two states (S1 Fig).

The positive slope must be equal to or larger than zero. In one situation, when two characters both have a positive slope in the same text snippet, then they both contribute to the average. In another situation, only one character has a positive slope in the same text snippet, the slope of the character without a positive slope will be counted as zero. So long as one of the two characters has a positive slope, both characters contribute to the average.

**S2 Fig. The Decrease in Happiness, Conditional on the Co-Occurrence with the Other Gender, is Higher for Female Than for Male Characters.** We analyze three datasets, including 6,087 movie synopses (**a**), 1,109 movie scripts (**b**), and 7,226 books (**c**). The decrease in happiness conditional on the co-occurrence with the other gender, measured in the average of negative regression coefficients *k*, are shown as bars (blue for males and orange for females). The lines on the bottom of the bars show one standard deviation. Asterisks indict P values. * P ≤ 0.05, **P ≤ 0.01, *** P ≤ 0.001, and ns non-significant. The result is significant across the three datasets.

**#1.4 - It would be beneficial to provide statistical significance for all comparisons in the paper (e.g., in Fig. 2, 3, 4, 7).**

We are regretting that we forgot to include the p-values. The current version has p-values for all comparisons. Asterisks indict P values. * *P* ≤ 0.05, ***P* ≤ 0.01, *** *P* ≤ 0.001, and ns non-significant.

**#1.5 - Fig. 2: “The increase in happiness is quantified by the weighted average of positive OLS regression coefficients” -> what are the weights? How do you get them?**

We apologize for the confusion caused by the wording. The sentence is reframed as: “The increase in happiness is quantified by the average of positive OLS regression coefficients, weighted by sample size (n of sentences in the regression). ”

Please see Methods (Measuring the Increase and Decrease in Happiness During the Co-Occurrence) for more information.

**#1.6 - Table 1: please provide more explanations. Do you fit one regression model with all these variables? What do these variables mean? What do the stars mean? The gender of the leading character has a big impact on both quality and popularity of the movie. It will be interesting to see the impact of emotional change separately on movies with a male or female leading character.**

We fitted two OLS linear regression models with all variables. One model is for predicting the rating of movies (quality); the other is to predict the number of votes of movies (popularity).

For dependent variables, rating means the scores that the audience rates of the movie, the range is 0-10, we assume the higher the score, the better the quality is. N of votes means the number of audiences who rate for the movie. We assume this number is a good proxy for the popularity of movies. For independent variables, the leading gender means the gender of the main character who dominantly show up in the movie script, where the male is 1 and female is 0. N of co-occurrence implies the number of sentences that the characters of both genders appear in the same sentence. Increase in the happiness of females is the mean value of positive slopes for females, while the increase in the happiness of males is the mean value of positive slopes for males.

We follow the suggestions of the reviewers to separate movies based on male and female leading character. The leading character is determined by the frequency of the name. We find that females are emotionally dependent on males in male-leading stories (the increase in happiness of females is higher than males when they co-occur), which account for a majority of all narratives (74% movies synopsis, 75% movies scripts). The result is significant across 6,087 movie synopses (a) and 1,109 movie scripts (b). While in female-leading movies, the dependency of females on males is not significant.

**#1.7 - Sec. 3, p. 6: “… we separate the movie synopsis data into female and male groups” -> How? Based on the gender of the main character?**

Yes, it is based on the gender of the leading character as noted in the legend of S4 Fig.

**#1.8 - Sec. 3, p. 6: why do you look for words “before and after the co-occurrence of characters to construct word co-occurrence network”? How do you separate which words describe which character? Why don’t you simply look at words around a single character (male or female)?**

The analysis presented in the last version was for unpacking the context in which characters of different genders meet. We agree with the reviewer that analyzing words around a single character serves better for the goal of revealing gender (Figure 4, 5 in the main body).

**#1.9 -The authors need to at least acknowledge that there are many sources of potential errors in their analysis pipeline, e.g.:**

**Automatic process of obtaining happiness scores is error-prone (correlation with manually obtained scores is 0.53);**

**Happiness scores of individual words can change dramatically in some contexts (e.g., in the presence of negation);**

**Automatic process of main character and their gender identification is error-prone.**

We fully agree that noting the methodological limitations is necessary and essential. We appreciate the reminding from the reviewer. We now acknowledge the boundaries of our methods in the discussion session.

**#1.10 It would be interesting to analyze gender stereotypes over time, especially in movies, to see if there have been any changes/improvements in the last years.**

We appreciate the reviewer for this great suggestion. We included this analysis in the current version (**S7 Fig)**. We find that gender stereotypes remain substantial, if not amplifying, over the years covered by our datasets.

**S7 Fig. The Increase in Happiness, Conditional on the Co-Occurrence with the Other Gender, is Higher for Female Than for Male Characters. This Finding is Robust across Time Periods.** We analyze three datasets, including 6,087 movie synopses (**a**), 1,109 movie scripts (**b**), and 7,226 books (**c**). The increase in happiness conditional on the co-occurrence with the other gender across different times, measured in the average of positive regression coefficients *k*, are shown as bars (blue for males and orange for females). The lines on the top of the bars show one standard deviation. Asterisks indict P values. * P ≤ 0.05, **P ≤ 0.01, *** P ≤ 0.001, and ns non-significant. The result is significant across the three datasets.

**Minor comments:**

**#1.11 - The paper needs thorough proof-reading.**

- The figures are of low quality and hard to read.

- P. 4: “Figure 7” -> “Figure 3”?

- Fig. 1: name highlighting is wrong.

- Fig. 5 and 6: legends are almost the same, need fixing. Fig. 6: “female” -> “male”? Also, 1,750 (female) + 4,337 (male) = 6,087 movie synopsis. What about the rest of 6,087 movie synopsis?

We proof-read the article thoroughly and included high-resolution figures. All these minor suggestions are incorporated in the current version gracefully. The missing movies are cartoons or movies in which only one gender of are identified, and now we change to 6,087 movie synopsis.

**Reviewer #2: The manuscript "The Cinderella Complex: Word Embeddings Quantify Gender Stereotypes in Movies and Books" examined whether and how much the so-called Cinderella complex is present in books and movie synopsis and scripts. Specifically, the authors aimed to identify how stories of movies and book contribute to perpetrate gender stereotypes. I think that this paper has many merits, in specific the authors conducted rigorous analyses of the texts that allowed to identify how stories contain a specific representation of women. However, I think that the manuscript needs to be deeply revised to improve its quality. In the following I will elaborate my major concerns.**

We thank the reviewer for noting the merits of our manuscript, especially our efforts in extensive text analysis. Following the insightful suggestions from the reviewer, we have thoroughly revised the manuscript. We believe the quality of the paper has been significantly improved.

**Introduction**

**#2.1 I have two major points that should be addressed in the introduction.**

**The first is related to gender stereotypes. They are simply cited with no explanation of what they actually are. Which are the contents of gender stereotypes and which are they functions? Social Psychological research have clearly shown that there are stereotypical expectations according to which women should be warm, communal, sociable, kind, etc, whereas men should be competent, agentic, skilled and assertive (for reviews see, Ellemers, 2018; Eagly, Wood, & Diekman, 2000). These stereotypes derive from the social roles historically attributed to men and women and contribute to maintain gender inequality in the society (especially in the workplace) at different levels. I think that this should be considered in the introduction and also in the general discussion to improve the broad quality of the paper. Indeed, gender stereotypes are very often mentioned by never explained deeply. Also, I wonder whether the Cinderella effect can be considered a stereotype. If yes (and I am not completely sure about it) the authors should motivate this possibility considering relevant scientific literature. In this vein, I would ask to motivate with more reference if the Cinderella complex could be considered a theory. Is this really a theory? For which scientific area? In the GD it should be also discussed how the Cinderella complex in movies and book can contribute to perpetrate gender stereotypes and inequality.**

We thank the reviewer for guiding our attention to relevant gender stereotype literature that anchors our study. We revised the introduction (Please kindly read the subsection titled Gender Stereotypes: Women’s Lacking of Competence and Agency) and discussion to unfold relevant literature.

We agree with the reviewer that the Cinderella complex is a social fact as evidence of gender stereotypes and not a theory itself. We explain in the current version that Cinderella complex, i.e., females’ hidden feelings of incompetence and psychological dependence (Dowling, 1981), is associated with the stereotypical expectations that women should be warm, communal, sociable, kind, etc, whereas men should be competent, agentic, skilled and assertive (for reviews see, Ellemers, 2018; Eagly, Wood, & Diekman, 2000, Saha, 2016). We also mentioned in the discussion that these stereotypes derive from the social roles historically attributed to men and women and contribute to maintaining gender inequality in the society (especially in the workplace) at different levels. Again, we thank the reviewer for helping us construct a more coherent and accurate narrative.

We added the following reference in the current version:

- Miller, J. B. (1986). What do we mean by relationships? Work in Progress No. 22. Wellesley, MA: Stone Center, Working Paper Series.
- Gilligan, C., Lyons, N. P., and Hanmer, T. J. (Eds.). (1990). Making connections. Cambridge, MA: Harvard University Press.
- Eagly, A. H., Wood, W., & Diekman, A. B. (2000). Social role theory of sex differences and similarities: A current appraisal. In T. Eckes & H. M. Traunter (Eds.), The developmental social psychology of gender (pp. 123–174). Mahwah, NJ: Lawrence Erlbaum.
- Saha, S., Safri, T. (2016), Cinderella Complex: Theoretical Roots to Psychological Dependency Syndrome in Women, International Journal of Indian Psychology, Volume 3, Issue 3, No. 8, DIP: 18.01.148/20160303, ISBN: 978-1-365-12176-0
- Ellemers, N. (2018). Gender stereotypes. Annu. Rev. Psychol. 69, 275–298.

**#2.2 Second, when presenting the study, the authors anticipate the results instead of clarifying the hypotheses of their study: “By analysing a large size of stories using word embeddings, we discovered that the so-called Cinderella complex widely exists in movies and novels. In the following sections, we first present our analysis on the case of Cinderella”. They should explicitly state what they expect from the specific analyses and why (the reasons behind these hypotheses clearly grounded on the scientific literature).**

We are very grateful to the reviewer’s suggestion. We explained the motivation of the current study with a literature context in the introduction to explicitly state three assumptions.

**Method**

**#2.3 The authors should provide more information on how the material has been selected. They wrote “rigorously” but with which criteria? I do not understand how they arrived at the number of 16255 movies. And from these only 6657 were selected with the criteria of the length of the synopsis? The same comment applies to books.**

We thank the reviewer for pointing this out, and we explained in the current version that the removals to secure data quality. We collect three datasets for this present research, including movie synopses, movie scripts, and books. To be specific:

First, we collect the movie synopsis data from the IMDB website ([www.imdb.com](http://www.imdb.com)). We select the movies with user ratings, plot synopsis, release year, and genre. And we get 16,255 movies for further data filtering. We choose 6,087 movie synopses with more than five sentences and both female and male characters in the analysis.

Second, we also collect the movie script data from the IMSDB website ([www.imsdb.com](http://www.imsdb.com)), which is the largest database of online movie scripts. There are 1,109 movie scripts after filtering out those in which only one gender of characters are identified. The metadata of the movie scripts, such as the release year and genre, is also collected.

Third, in addition to the two movie datasets, we also collect the data of more than 40 thousand English books from the Gutenberg Project ([www.gutenberg.org](http://www.gutenberg.org/)), including the text of story, publication time, and genre. In the data filtering of books, only 7,226 books belonging to the genre "language and literature" and containing both female and male characters are selected.

**#2.4 As far as the “fortune” measure, did they use only the words cited in the text (i.e., success, happy, lucky, and failure, sad, unlucky)? If this is the case, this should be extensively motivated, because many other words could be related to the “fortune” construct. Importantly, why “fortune”? This should be anticipated in the theoretical part of the paper. How the aim of the study to look for the Cinderella complex in story telling is achieved by analysing the “fortune” of the character? This is a major point that the authors should address.**

We thank the reviewer’s comments, which urged us to strengthen connections between theories and empirical analysis. Since our key theoretical concern is the emotional dependency of females on males, we use sentiment analysis based on word embeddings to measure the happiness scores for female and male characters over time. To avoid unnecessary misunderstandings on the word of fortune, we use the term happiness vector instead.

We explained in the current version that the construction of happiness vector using two groups of positive and negative words(success, succeed, lucky, fortune, happy, glad, joy, smile for positive and failure, fail, unfortunate, unhappy, sad, sorrow, tear for negative) was not only theoretically motivated (Vonnegut, 1981, Dodds, 2016), but also a practice of established methods to construct the cultural dimension (the construction of cultural dimension in [Caliskan](http://paperpile.com/b/ynVwMM/Pmqt) et al, 2017; Garg et al, 2017; Kozlowski et al, 2018). We also ran robustness tests using other words and found the findings remained unchanged. To be specific, we test the validity of this method by computing the Pearson correlation coefficients between two versions of happiness scores, one estimated from the happiness vector and the other labeled using Amazon’s Mechanical Turk service. The correlation coefficient is 0.53 (p<0.001) across the top 10,000 popular words, justifying the construction of the fortune vector as a measure of happiness.

**#2.5 Why does the first study compare Cinderella and Forrest Gump? How Forrest Gump was chosen? It seems to me that they are not comparable for many reasons.**

We agree that the Forrest Gump example is distracting. It is removed from the current version.

**Results**

**#2.6 The regression table is not clear to me. Beta, F and p values are missing. What are the values in parentheses? Moreover, how many models were run? Moreover, I do not fully understand why the result showing a higher relation between happiness of women and popularity could be interpreted as in line with the Cinderella complex or at least the fact that gender stereotypes are appreciated. This should be clearer commented.**

The statistics suggested by the reviewer are included. We fitted two OLS linear regression models. One predicts the rating of movies (quality), the other predicts the number of votes of movies (popularity). The values in parentheses were standard errors. In this new version of the manuscript, we remove the standard errors in parentheses to avoid misunderstandings. For p values in the table. * means the p values is between 0.01-0.05, ** mean between 0.001-0.01, *** means <0.001. Beta is the value before *, And we added one row to Table 1 to show the F-statistic.

Theoretically, the social and cultural roots of gender stereotypes form social force against stereotype disconfirmation from people, action, or ideas (Ellemers, 2018). In this sense, the stories that approve gender stereotypes will gain social approval themselves, whereas the stories against stereotypes will be ignored and disapproved. Increase in happiness for female means that when they meet male characters, they feel happier, which reflects female emotionally depends on males. A higher relation between the increase in happiness and popularity means people like movies in which female depends on males emotionally more. It suggests gender stereotypes or stories with Cinderella complex are appreciated by people.

The Cinderella complex is more directly associated with Figure 3. The regressions in Table 1, which show that the stronger presentation of Cinderella complex is related with higher popularity of movies, are suggestive evidence for the underlying social mechanism reproducing the Cinderella complex in particular, and gender stereotypes in general. We thank the reviewer for reminding us to differentiate these two parts, which are elaborated separately in the current version.

- Ellemers, N. (2018). Gender stereotypes. Annu. Rev. Psychol. 69, 275–298.

**#2.7 It could be interesting also examining the relation between the number of co-occurrences and increase happiness.**

We explored along the direction as suggested by the reviewers and found that the increase in happiness, either for female or male, is negatively correlated with the number of co-occurrence. The correlation coefficients are -3.80 (p<0.001) and -3.89 (p<0.001), respectively.

The comment from the reviewer reminded us to test the collinearity between these two variables in the regressions. We ran stepwise regressions to control one variable at a time and found that the number of co-occurrence does not rule out the impact of happiness increase on the size and scale of movie rates.

**Table 2. OLS Linear regression models predicting the quality of movie synopsis.** For p values, * denotes 0.01-0.05, ** denotes 0.001-0.01, and *** denotes <0.001.

|  | **Quality (rating)** | | |
| --- | --- | --- | --- |
| Constant | 6.21*** | 6.27*** | 6.23*** |
| Leading gender (male=1, female=0) | 0.19*** |  |  |
| N of co-occurrence |  | 0.01*** |  |
| Increase in happiness for the female |  |  | 0.06*** |
| Increase in happiness for the male |  |  | -0.11*** |
| R squared | 0.005 | 0.004 | 0.010 |
| F-statistic | 31.78 | 26.76 | 15.90 |
| N of cases | 6,087 | | |

**Table 3. OLS Linear regression models predicting the popularity of movie synopsis.** For p values, * denotes 0.01-0.05, ** denotes 0.001-0.01, and *** denotes <0.001.

|  | **Popularity (N of votes)** | | |
| --- | --- | --- | --- |
| Constant | 8.55*** | 8.29*** |  |
| Leading gender (male=1, female=0) | 0.39*** |  |  |
| N of co-occurrence |  | 0.06*** |  |
| Increase in happiness for female |  |  | 0.06* |
| Increase in happiness for male |  |  | -0.39*** |
| R squared | 0.004 | 0.05 | 0.03 |
| F-statistic | 25.56 | 341.5 | 56.11 |
| N of cases | 6,087 | | |

**Discussion**

**#2.8 The specific and general implications of the results are poorly discussed. For instance, why books and movies with male characters include more verbs than those with female main characters? Here I would like to see a discussion concerning, for example, the agency characteristics stereotypically attributed to men and this result. Moreover, why adjectives are more represented for female characters? How this result could be interpreted? One possibility is that women are described in more abstract terms related to their traits and therefore are represented in a more complex way than men? Moreover, adjectives lead to inferences of stability of these traits, which are perceived as difficult to change (See Maass, 1999). In this line of reasoning, which are the implications for gender stereotypes?**

We appreciate the reviewer’s suggestion to unfold the implications of our results. We revisited the gender stereotype theories in the section of the **introduction** and explained that our results on verbs highlight the stereotypical expectations that men are more agentic and active and women are more communal and passive (Ellemers, 2018, Eagly, Menegatti & Rubini, 2017). Indeed, men are active and subjective, whereas women are passive and objective; men give, and women take in relationships (Graumann & Theo, 1989, p95-96).

We thank the reviewer for suggesting the research of (Maass, 1999). We have tried to examine the distribution of adjectives across genders further. And we find that females are more likely to be described as “beautiful”, while males are more likely to be described as “able”. However, we are regretting that there is no significant difference between the number of adjectives used by females compared with males. We have discussed the potential theoretical contributions in the section of Discussion and conclusions.

**#2.9 At the beginning of the manuscript, the authors write about moral tales. Could this argument be discussed in the GD in order to explain how morality is related to gender stereotypes and the role of movies and book to perpetrate gender inequality though these morality norms?**

We thank the reviewer for this great suggestion. We added in the discussion on how gender stereotypes, constructed and weaved into the moral tales from movies and books, may maintain gender inequality though these morality norms and reproduce gender inequality as a social fact (Narahara, 1998; Bussey & Bandura, 1999). For example, when children are exposed to stereotyped narratives, they may fill themselves into stereotypical roles [(Narahara, 1998)](https://paperpile.com/c/ynVwMM/i6yq). As shown in a study on the impact of Disney movies [(Bazzini, Curtin, Joslin, Regan, & Martz, 2010)](https://paperpile.com/c/ynVwMM/dTAi), children who rated beautiful characters more favorably than less beautiful characters tend to apply the same principle in daily life.

**#2.10 Finally, it should be interesting some comments related to language use and the content of gender stereotypes, that is how language contribute to the maintenance of these stereotypes (see Menegatti and Rubini, 2017).**

We thank the reviewer for pointing our attention to this apparently relevant literature. We cited and discussed this reference in the current version.

**#2.11**

**Minor points:**

**The numbers of pages are missing, they should have been added to facilitate the reading and revision. Manuscript text should also be double-spaced.**

**The manuscript ends with “etc”. This should be avoided.**

**References**

1. **Eagly, A. H., Wood, W., & Diekman, A. B. (2000). Social role theory of sex differences and similarities: A current appraisal. In T. Eckes & H. M. Traunter (Eds.), The developmental social psychology of gender (pp. 123–174). Mahwah, NJ: Lawrence Erlbaum.**
2. **Ellemers, N. (2018). Gender stereotypes. Annu. Rev. Psychol. 69, 275–298.**
3. **Maass, A. (1999). Linguistic intergroup bias: Stereotype perpetuation through language. In M. P. Zanna (ed.), Advances in Experimental Social Psychology (Vol. 31, pp. 79121). San Diego, CA: Academic Press.**
4. **Menegatti, M. & Rubini, M. (2017). Gender bias and sexism in language. In Oxford Research Encyclopedia of Communication. Oxford University Press.**

All these points are checked off in the current version. In all, we appreciate the reviewers’ constructive and inspiring comments, from which we have benefited a lot. We appreciate the reviewer’s help, and we believe the quality of the manuscript has been greatly improved.
