# Supplementary material for: The Cinderella Complex: Word embeddings reveal gender stereotypes in movies and books
Source: PLoS One. 2019 Nov 22;14(11):e0225385. doi: 10.1371/journal.pone.0225385 (PMC6874350; doi:10.1371/journal.pone.0225385)
Supplement: Attachment — Submitted filename: Response to Reviewers.docx [file pone.0225385.s011.docx]

**Response Letter**

Dear Dr Wang,

Thank you for submitting your manuscript to PLOS ONE. After careful consideration, we feel that it has merit but does not fully meet PLOS ONE’s publication criteria as it currently stands. Therefore, we invite you to submit a revised version of the manuscript that addresses the points raised during the review process.

We would appreciate receiving your revised manuscript by Dec 01 2019 11:59PM. When you are ready to submit your revision, log on to https://www.editorialmanager.com/pone/ and select the 'Submissions Needing Revision' folder to locate your manuscript file.

If you would like to make changes to your financial disclosure, please include your updated statement in your cover letter.

To enhance the reproducibility of your results, we recommend that if applicable you deposit your laboratory protocols in protocols.io, where a protocol can be assigned its own identifier (DOI) such that it can be cited independently in the future. For instructions see: <http://journals.plos.org/plosone/s/submission-guidelines#loc-laboratory-protocols>

Please include the following items when submitting your revised manuscript:

A rebuttal letter that responds to each point raised by the academic editor and reviewer(s). This letter should be uploaded as separate file and labeled 'Response to Reviewers'.

A marked-up copy of your manuscript that highlights changes made to the original version. This file should be uploaded as separate file and labeled 'Revised Manuscript with Track Changes'.

An unmarked version of your revised paper without tracked changes. This file should be uploaded as separate file and labeled 'Manuscript'.

Please note while forming your response, if your article is accepted, you may have the opportunity to make the peer review history publicly available. The record will include editor decision letters (with reviews) and your responses to reviewer comments. If eligible, we will contact you to opt in or out.

We look forward to receiving your revised manuscript.

Kind regards,

Ilya Safro, Ph.D.

Academic Editor

PLOS ONE

Dear Dr. Ilya Safro,

We are grateful for the opportunity to revise the manuscript in response to these thoughtful reviews, and we believe that the paper is much stronger for having incorporated the suggestions from the reviewers. We detail our responses and revisions as below.

[Note: HTML markup is below. Please do not edit.]

Reviewers' comments:

Reviewer's Responses to Questions

Comments to the Author

1. If the authors have adequately addressed your comments raised in a previous round of review and you feel that this manuscript is now acceptable for publication, you may indicate that here to bypass the “Comments to the Author” section, enter your conflict of interest statement in the “Confidential to Editor” section, and submit your "Accept" recommendation.

Reviewer #1: (No Response)

Reviewer #2: All comments have been addressed

2. Is the manuscript technically sound, and do the data support the conclusions?

The manuscript must describe a technically sound piece of scientific research with data that supports the conclusions. Experiments must have been conducted rigorously, with appropriate controls, replication, and sample sizes. The conclusions must be drawn appropriately based on the data presented.

Reviewer #1: Yes

Reviewer #2: Yes

3. Has the statistical analysis been performed appropriately and rigorously?

Reviewer #1: Yes

Reviewer #2: Yes

4. Have the authors made all data underlying the findings in their manuscript fully available?

The [PLOS Data policy](http://www.plosone.org/static/policies.action#sharing) requires authors to make all data underlying the findings described in their manuscript fully available without restriction, with rare exception (please refer to the Data Availability Statement in the manuscript PDF file). The data should be provided as part of the manuscript or its supporting information, or deposited to a public repository. For example, in addition to summary statistics, the data points behind means, medians and variance measures should be available. If there are restrictions on publicly sharing data—e.g. participant privacy or use of data from a third party—those must be specified.

Reviewer #1: Yes

Reviewer #2: Yes

5. Is the manuscript presented in an intelligible fashion and written in standard English?

PLOS ONE does not copyedit accepted manuscripts, so the language in submitted articles must be clear, correct, and unambiguous. Any typographical or grammatical errors should be corrected at revision, so please note any specific errors here.

Reviewer #1: Yes

Reviewer #2: Yes

6. Review Comments to the Author

Please use the space provided to explain your answers to the questions above. You may also include additional comments for the author, including concerns about dual publication, research ethics, or publication ethics. (Please upload your review as an attachment if it exceeds 20,000 characters)

Reviewer #1: I would like to thank the authors of the manuscript for their substantial efforts at addressing all the reviewers’ concerns. I found the additional experiments presented in Figures S1-S6 very helpful. I believe their inclusion into the manuscript will strengthen the paper. Unfortunately, right now they are left only as figures in the Supplement with no explanation and no reference in the main body. It would be great to include these experiments and describe what was done, why, and what conclusions can be drawn from them in the main body of the paper or at least in the Supplemental Materials. Also, the authors provided to the reviewers detailed explanations for the experiments, but not all of these explanations found their way into the main manuscript. In particular, I think it is important to mention that happiness score normalization is done within a character, and what experiments were performed to confirm robustness of the results in Table 1 to story intensity and the gender of the leading character.

We appreciate the reviewer for these great suggestions. We now discuss additional experiments in the main body and supporting information file as suggested. In particular, 1) we mention the happiness score normalization within a character in the method part (p. 22), and 2) explain the robustness test against story intensity and the gender of the leading character (p. 13).

There are still some missing details:

- “We accumulate the happiness curve across sentences or paragraphs …” (p. 20) and “We then sum the averaged happiness scores across sentences … to obtain the emotion curves” (p. 6). What do you mean by “accumulate the curve”? Do you really sum the scores across sentences or do you just plot the scores for all the sentences? Do you do any sort of curve smoothing?

- Fig. 8a: Why are the frequency numbers (y axis) less than 1 (negative powers of 10) for movie synopsis?

We regret that we did not explain the method for drawing curves more clearly, and we appreciate the comments from the reviewer, which provided an opportunity for us to unfold measure explanation.

The emotional status of characters is built up by a sequence of events; therefore, we sum the scores across sentences alongside the story timeline to measure the cumulative average rather than the marginal variance of emotion. For example, in the story of Cinderella (Fig 1b), the lowest point of the emotional curve of Cinderella (at around 30 percentile of the story timeline) is the consequence of the death of parents and maltreatment from the stepmother. Therefore, the emotional status of Cinderella at this stage is better measured by the cumulative score rather than the score of any single sentence. We did not do curve smoothing.

The above text is included in the current version (p. 6).

We thank the reviewer for pointing out the mistake of Figure 8a. In earlier versions, we calculated probability but displayed the y-axis label as frequency. We have corrected it in this version.

Phrasing:

- “… men are more likely to use verbs than women” (p. 5) and “Males use more verbs than females” (Fig. 6). I think you mean that men are more likely than women to be described using verbs.

We thank the reviewer for providing a better and more precise expression. We have included it in the current version.

Reviewer #2: (No Response)

7. PLOS authors have the option to publish the peer review history of their article (what does this mean?). If published, this will include your full peer review and any attached files.

If you choose “no”, your identity will remain anonymous but your review may still be made public.

Do you want your identity to be public for this peer review? For information about this choice, including consent withdrawal, please see our Privacy Policy.

Reviewer #1: No

Reviewer #2: Yes: Michela Menegatti

[NOTE: If reviewer comments were submitted as an attachment file, they will be attached to this email and accessible via the submission site. Please log into your account, locate the manuscript record, and check for the action link "View Attachments". If this link does not appear, there are no attachment files to be viewed.]

While revising your submission, please upload your figure files to the Preflight Analysis and Conversion Engine (PACE) digital diagnostic tool, <https://pacev2.apexcovantage.com/>. PACE helps ensure that figures meet PLOS requirements. To use PACE, you must first register as a user. Registration is free. Then, login and navigate to the UPLOAD tab, where you will find detailed instructions on how to use the tool. If you encounter any issues or have any questions when using PACE, please email us at figures@plos.org. Please note that Supporting Information files do not need this step.

In compliance with data protection regulations, you may request that we remove your personal registration details at any time. [(Remove my information/details)](https://www.editorialmanager.com/pone/login.asp?a=r). Please contact the publication office if you have any questions.
